# High-Performance Ethylene Glycol Sensor Based on Imine Covalent Organic Frameworks

**DOI:** 10.3390/nano13243103

**Published:** 2023-12-08

**Authors:** Shiwei Liu, Guojie Zhang, Weiyu Zhang, Ning Tian, Qihua Sun, Zhaofeng Wu

**Affiliations:** 1 Xinjiang Key Laboratory of Solid-State Physics and Devices, Urumqi 830046, Chinasunqh@xju.edu.cn (Q.S.); 2 School of Physics Science and Technology, Xinjiang University, Urumqi 830046, China

**Keywords:** ethylene glycol sensor, covalent organic frameworks, selectivity, long-term stability

## Abstract

The colorless and odorless ethylene glycol is prone to unknowingly causing poisoning, making preventive monitoring of ethylene glycol necessary. In this paper, scandium (III) trifluoromethanesulfonate was used as a catalyst to successfully prepare covalent organic framework (COF) nanospheres linked by imines at room temperature. The COF nanospheres were characterized by XRD, SEM, TEM, FT-IR, UV-Vis and BET. The results show that COF nanospheres have rough surfaces and a large number of mesoporous structures, which greatly increase the active sites on the surface of the sensing material and enhance the gas sensing performance. The sensing results showed that the prepared imine-conjugated COF nanospheres exhibited a good response–recovery ability for 10 consecutive response–recovery cycles for ethylene glycol at room temperature and had a theoretical detection limit of 40 ppb. In addition, the responses of COF nanospheres to nearly 20 interfering gases, including HCl, HNO_3_, phenol, formaldehyde and aniline, are relatively low compared to the response to ethylene glycol, indicating that the COF nanospheres have high selectivity towards ethylene glycol. The COF nanospheres show good sensitivity and selectivity for the detection of ethylene glycol, which should be attributed to the large specific surface area, hydrogen bonding interactions, and high defects. This work provides an effective method for the detection of ethylene glycol and expands the application field of COF materials.

## 1. Introduction

Ethylene glycol (C_2_H_6_O_2_) is a widely used organic chemical, often used as an organic solvent in automotive antifreeze and in paints and coatings. C_2_H_6_O_2_ is highly toxic and lethal, mainly because of its colorless and odorless nature, which can lead to unintentional inhalation and subsequent difficulty in determining the cause of poisoning [1]. If left untreated after inhalation, C_2_H_6_O_2_ is metabolized to glycolic acid and oxalic acid, leading to metabolic acidosis, acute renal failure and death [2,3]. In addition, C_2_H_6_O_2_ is highly flammable, and its vapors readily ignite or detonate when exposed to open flames [4], making the development of high-performance C_2_H_6_O_2_ sensors critical to protecting individuals from its hazards.

Therefore, preventive detection of C_2_H_6_O_2_ is necessary and important for the prevention of poisoning and combustion. Currently, commonly used methods for C_2_H_6_O_2_ detection include gas chromatography [5], spectrophotometry [6] and flame ionization detection [7]. However, due to the complexity of these methods and the inability to detect C_2_H_6_O_2_ in real time, they can only be used as a means of chemical analysis and detection for gas detection and monitoring [8,9]. As an important device for detecting and measuring the type and concentration of gases in the environment, gas sensors are characterized by simple operation and short detection time compared to other gas detection methods [10,11,12]. For example, a variety of gas sensors have been developed for the detection of C_2_H_6_O_2_: Liu et al. prepared ZnO/ZnCo_2_O_4_ composites using a one-step hydrothermal method, which showed a high response to, and excellent selectivity for, C_2_H_6_O_2_ at 160 °C [13]. Ding et al. prepared ZnO/rGO nanosheets using chemical precipitation and hydrothermal methods and obtained the best gas sensitivity through high-temperature annealing treatment at 220 °C. The best gas sensitivity was obtained at 220 °C [14]. However, these C_2_H_6_O_2_ sensors need to be at high temperatures for optimal performance [15], which itself carries a certain degree of danger [4]. Therefore, there is an urgent need for an ambient-temperature C_2_H_6_O_2_ sensor.

Covalent organic frameworks (COFs) are a new class of crystalline porous materials consisting of light elements bonded by strong covalent bonds [16,17]. COFs have many unique properties, such as π-π conjugated structure, good electrical conductivity and large specific surface area, and the various functional groups and chemical bonds present in the backbones of the COFs provide a rich variety of active sites. Recently, COFs have been widely used in many fields [18,19]. COFs are also used for gas-sensitive detection; for example, Choi et al. combined COFs with rGO to achieve selective detection of NO_2_ [20]. Krishnaveni et al. hybridized Pd NPs with imine-based covalent organ skeletal nanosheets (ePd@TpPa-SO_3_H COFs) to achieve high-performance detection of H_2_ [21], which greatly expanded the application areas of COFs. However, there are no COFs available for C_2_H_6_O_2_ detection.

Preventive detection of C_2_H_6_O_2_ is important as it is a VOC that can easily cause poisoning. In this work, COF nanospheres with rough surfaces and a large number of pore structures were successfully prepared by using scandium (III) trifluoromethanesulfonate (Sc(OTf)_3_) as a catalyst to promote the synthesis of imine bonding between monomers via a dehydration reaction under room-temperature conditions. The high sensitivity and good selectivity of the COF nanosphere sensors for C_2_H_6_O_2_ at room temperature were attributed to their large specific surface area, pore structure and abundant functional groups, which added a large number of active sites for gas adsorption.

## 2. Materials and Methods

### 2.1. Materials and Reagents

The materials and reagents involved in this work are described in the Supplementary Materials.

### 2.2. Preparation of Materials

First, 70.3 mg of 1,3,5-tris(4-aminophenyl) benzene (TAPB) and 63.06 mg of 4,4′-biphenyldicarbaldehyde (BPDA) were added to an 8 mL mixture of 1,4-dioxane and 1,3,5-trimethylbenzene (4/1, *v*/*v*) in a centrifuge tube and sonicated immediately until the monomers were completely dissolved. This mix resulted in the formation of a yellow solution. Next, 17.7 mg of catalyst Sc(OTf)_3_ was weighed and added to the resulting mixed solution, which was immediately covered and shaken vigorously to thoroughly mix the catalyst and mixed solution, producing a large amount of red precipitate during shaking. The mixture was incubated at room temperature for 72 h, during which the red precipitate gradually formed a spongy shape and deepened in color. The precipitate was collected by centrifugation and repeatedly washed and centrifuged with methanol (CH_3_OH) 5 times to remove unreacted monomers and catalysts. Finally, the precipitate was dried overnight in a vacuum drying oven at 70 °C. Eventually, a red COF powder was obtained, hereafter referred to as COF_TB_.

### 2.3. Sensor Preparation and Gas-Sensitive Testing

Sensor preparation: The previously prepared COF_TB_ was put into a mortar and ground into powder. A certain amount of acetonitrile was added to the mix and sonicated for 5 min. After waiting for the excess COF_TB_ to precipitate, the light-red solution of the upper layer was collected. The solution was dripped onto the silver interdigital electrodes and dried, forming a reddish COF_TB_ film on the electrode. The interdigital electrodes have a 13 mm × 7 mm × 0.5 mm alumina ceramic substrate with 5 pairs of silver fork fingers with a spacing of 200 μm between two fingers. After the sensor was prepared, it was stored at room temperature for about 24 h before use.

Gas sensitivity test: The gas sensitivity test was performed using a static test method [22], using a multi-function probe station (CGS-MT, Beijing, China), with a test voltage of 4 V. In order to minimize the interference of external environmental influences on the tests, the following methods were used to prepare the experimental gases: two clean experimental vessels of the same volume were filled with the same air to obtain the same initial environment, after which one vessel was sealed directly as the comparison air, and the other was added with the test liquid to be used as the target gas after the liquid was evaporated. The response is defined as response = (I_gas_ − I_air_)/I_air_ × 100%, where I_gas_ and I_air_ are the current of the sensor in the comparison air and target gas, respectively. The response time is defined as the time to reach 90% of the stable response value, and the recovery time is defined as the time to reach within 10% of the initial response value.

### 2.4. Characterization of Materials

The morphology of the samples was characterized by scanning electron microscopy (SEM, Thermo Fisher, Quattro S, Waltham, MA, USA) and transmission electron microscopy (TEM, JEOL, JEM 2100 F, Tokyo, Japan). The structure and composition of the samples were characterized using X-ray diffraction (XRD, Ultima, UltimaIV, Tokyo, Japan) and Fourier-transform infrared spectroscopy (FT-IR, Bruker, VERTEX 70 RAMI, Ettlingen, Germany). The absorbance of the samples was determined using ultraviolet–visible absorption spectroscopy (UV-Vis, PerkinElmer, Lambda 650, Waltham, MA, USA). Specific surface area was measured by using a multi-station extended automatic surface area and porosity analyzer (Micromeritics, ASAP 2460, Norcross, GA, USA).

## 3. Results and Discussion

### 3.1. Structure and Composition of the COF_TB_ Sample

**Figure 1:**
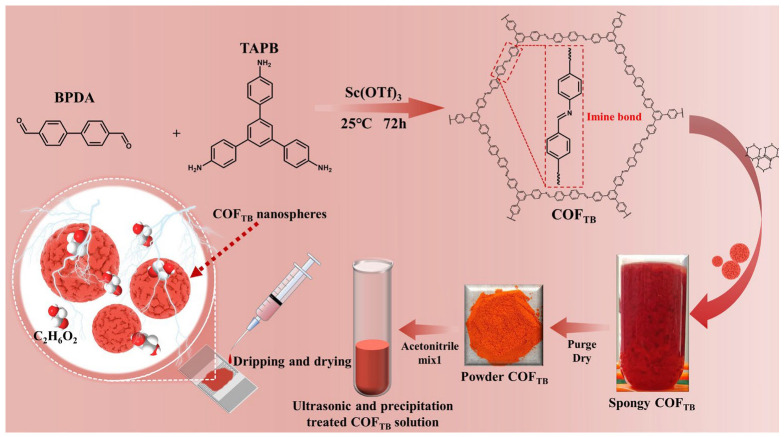
The preparation process of the COF_TB_ sensor.

For the synthesis of COF_TB_, the first step is to weigh 0.1 mmol of the two monomers and dissolve them in a mixture of 1,4-dioxane and trimethylbenzene (4/1, *v*/*v*). During this step, it is necessary to pay attention to the inside of the container at all times to prevent the monomers from forming a precipitate in solution, and to sonicate the two monomers until they are completely dissolved immediately upon their addition to the mixture. Errors in this step can cause inconsistency in the color of the synthesized material. Next, the catalyst was added to the mixture, and the container was immediately capped and shaken vigorously to fully mix the catalyst and the mixture solution, which was then incubated at room temperature for 72 h. Sc(OTf)_3_ acts as a Lewis acid to catalyze the synthesis of imine bonds during the reaction and has a higher catalytic efficiency compared to the use of acetic acid as a catalyst for the synthesis of COFs, and an excess of Lewis acid inhibits the exchange of imine bonds [23,24]. At the end of the reaction, the products were separated by centrifugation, and the precipitate was washed with methanol five times in order to remove unreacted monomer and catalyst that could interfere with subsequent characterization and testing. To prepare the sensor, the synthesized material was dissolved in acetonitrile solution, and the upper layer of the solution was collected and drop coated to ensure that the COF film on the sensor was of uniform thickness. Finally, the sensor was left for a period of time before use (Figure 1).

**Figure 2:**
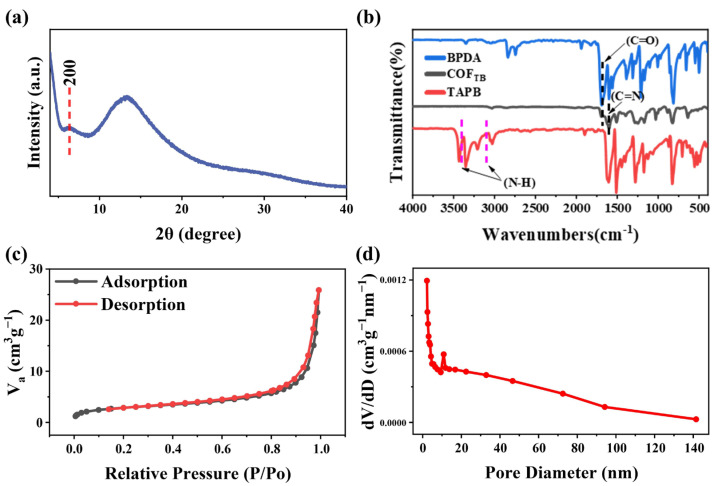
(**a**) XRD patterns of COF_TB_. (**b**) FT-IR spectra of COF_TB_. (**c**) N_2_ adsorption/desorption isotherms and (**d**) pore size distribution of COF_TB_.

The XRD spectrum of COF_TB_ is shown in Figure 2a, in which a few diffraction peaks can be observed, including a diffraction peak corresponding to the (200) crystalline plane. This peak indicates that the crystallite of the synthesized material is poor [25], suggesting that the rapid and large generation of the imine bond during the synthesis process leads to the disordered structure of the material. The functional groups of COF_TB_ were next analyzed using FT-IR (Figure 2b), and the stretched vibrational band located at 1617 cm^−1^ [26] was attributed to C=N, confirming the successful synthesis of the imine bond. There were no characteristic peaks of N-H observed in the range of 3100–3400 cm^−1^ [27] in the infrared spectra, suggesting that the obtained COF_TB_ lacked the presence of unreacted amino groups at the edge. Furthermore, a weak vibration of C=O was observed at 1697 cm^−1^ [28], which was attributed to the presence of an unreacted aldehyde end group at the edge of COF_TB_.

Next, to gain a more comprehensive understanding of the specific surface area and pore size distribution of the specimen, N_2_ adsorption/desorption experiments were conducted on the material at a temperature of 77 K. The Brunauer–Emmett–Teller (BET) curve (Figure 2c) of the material is a typical type II curve, reflecting that the adsorption process of COF_TB_ is a physical adsorption process of non-porous or microporous adsorbent. This indicates that COF_TB_ has a large pore size, which may be due to the pleats on the surface of the COF_TB_ nanosphere and the pore holes formed by the nanosphere stacking [29]. The NLDFT/GCMC method was then used to analyze the pore size distribution curve of the sample (Figure 2d). It can be seen that the sample has a wide range of pore size distribution, with a large number of mesoporous and microporous structures. The calculated specific surface area of COF_TB_ was 10.04 m^2^g^−1^ and the average pore size was 19.8 nm.

**Figure 3:**
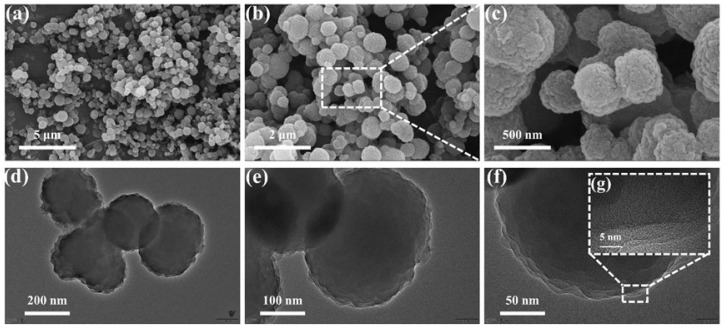
(**a**–**c**) SEM and (**d**–**g**) TEM of COF_TB_.

In order to understand the effect of material morphology on gas-sensitive properties, the morphological structure of the samples was analyzed using SEM and TEM. The SEM images of the material are shown in Figure 3a–c, demonstrating that COF_TB_ is composed of nanoparticle agglomerates with a particle size of about 500 nm, which form many channel structures. Further magnification shows that COF_TB_ has a rough surface which increases the reaction area of the material, is conducive to the adsorption of the gas molecules on the surface and improves the gas sensing performance [30,31,32]. Figure 3d–f show the TEM images of the material, in which it can be seen that the COF_TB_ material is a solid spherical structure and there are multiple spheres stacked together. Figure 3g shows that the COF_TB_ material has a highly disordered texture, which suggests that the material has an amorphous structure. This same conclusion was derived from the XRD results [33].

### 3.2. Gas Sensitivity of COF_TB_

**Figure 4:**
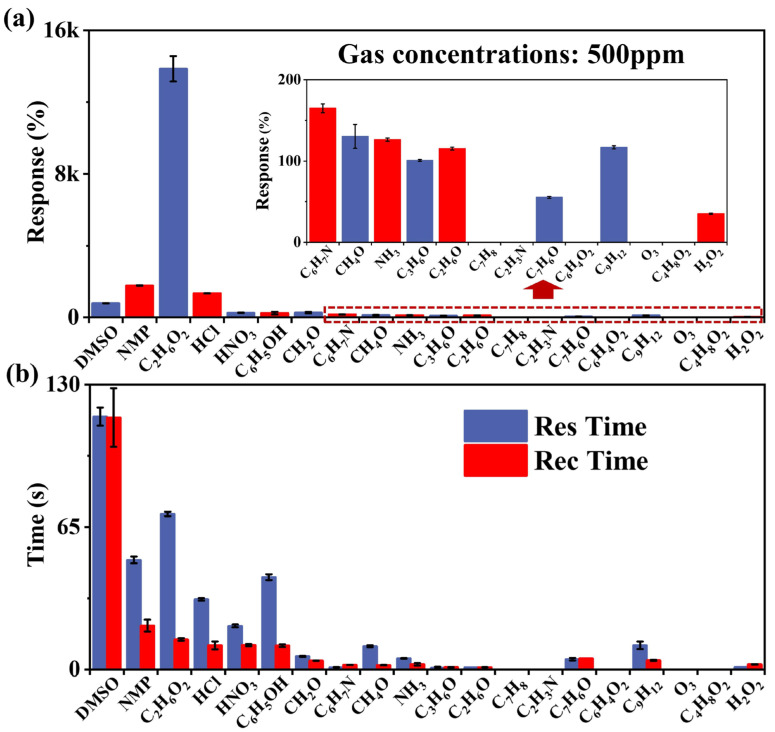
(**a**) Histograms of the magnitude of the response of COF_TB_ to different gases (500 ppm), and (**b**) histograms of the response time and recovery time to different gases.

Next, the gas-sensitive properties of COF_TB_ were investigated. The response value, response time and recovery time of COF_TB_ to C_2_H_6_O_2_ and 19 other gases (including DMSO, NMP, HCl, HNO_3_, C_6_H_6_O, CH_2_O, C_6_H_7_N, CH_4_O, NH_3_, C_3_H_6_O, C_2_H_6_O, C_7_H_8_, C_2_H_3_N, C_7_H_6_O, C_6_H_4_O_2_, C_9_H_12_, O_3_, C_4_H_8_O_2_, H_2_O_2_) at room temperature were compared. Figure 4a shows a histogram of the response size of COF_TB_ to different gases, showing that the response value of C_2_H_6_O_2_ is 13.9k%, or more than 7.8-times that of other gases, reflecting the better selectivity of the COF_TB_ gas sensor. Figure 4b shows a histogram of the response time and recovery time: the response time of C_2_H_6_O_2_ is 71 s, which is slower due to the fact that the adsorbed oxygen on the surface is not enough to oxidize the adsorbed C_2_H_6_O_2_, while the recovery time is 13.7 s. Figure S2 shows three response cycles of the COF_TB_ sensor for 20 different atmospheres, including C_2_H_6_O_2_. In summary, it can be seen that the COF_TB_ sensor has good selectivity in detecting C_2_H_6_O_2_. As shown in Figure S2, when the sensor is exposed to a reducing gas (such as CH_4_O, NH_3_ and C_3_H_6_O), the current rises rapidly, indicating a decrease in the resistance of the sensor. Reducing gases provide electrons to the COF_TB_ sample, and the decrease in resistance when the sensor is exposed to reducing gases suggests that COF_TB_ has n-type semiconducting properties [25].

**Figure 5:**
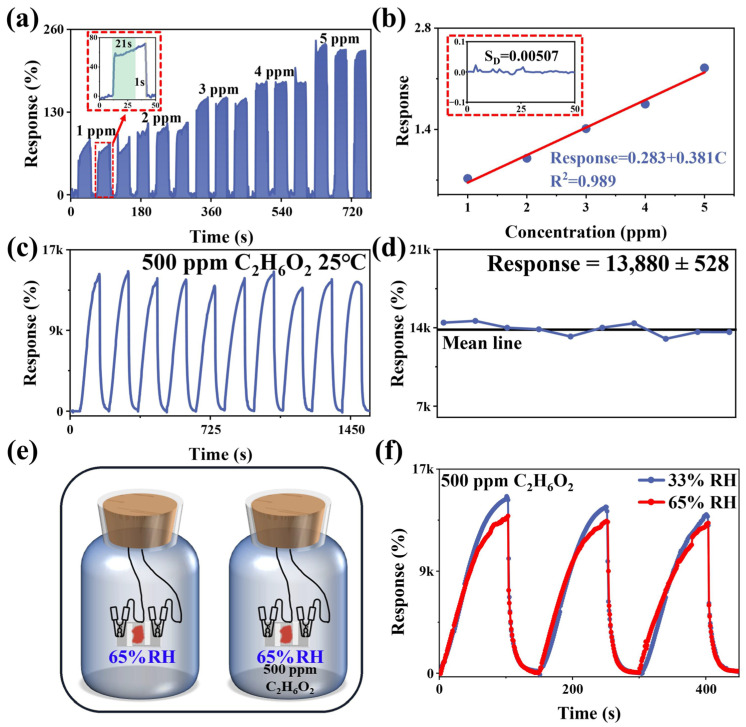
(**a**) COF_TB_ sensing curves for different concentrations (1–5 ppm) of C_2_H_6_O_2_ atmospheres at room temperature, and the insert is amplification of a response-recovery cycle; (**b**) linear fit between response values and C_2_H_6_O_2_ concentration, and the insert is standard deviation diagram; (**c**) ten response-recovery periods and (**d**) fluctuations of response value; (**e**) schematic of COF_TB_ testing at 65% RH for 500 ppm C_2_H_6_O_2_; (**f**) response curves to 500 ppm C_2_H_6_O_2_ at 33% RH and 65% RH, respectively.

In order to further evaluate the theoretical limit of detection (LoD) of the samples, the sensing curves were tested for different concentrations of C_2_H_6_O_2_ (Figure 5a), and a histogram of averages and error bars is illustrated in Figure S3. It can be seen that the magnitude of the response of C_2_H_6_O_2_ is positively correlated with the concentration of C_2_H_6_O_2_. The linear relationship between the response of the samples and the concentration is shown in Figure 5b. The response value of COF_TB_ is linear with the magnitude of the concentration of C_2_H_6_O_2_ at concentrations ranging from 1 to 5 ppm. This is based on LoD = 3S_D_/m, where S_D_ is the standard deviation of the noise in the response curve with a magnitude of 0.00507, and m is the slope of the linearly fitted curve with a magnitude of 0.381. Based on these calculations, the LoD of the COF_TB_ is about 0.04 ppm, which indicates that the COF_TB_ has a high sensitivity to C_2_H_6_O_2_. The insert in Figure 5a shows the response–recovery time of COF_TB_ for 1 ppm C_2_H_6_O_2_: the response time is 21 s and recovery time is 1 s. Figure 5c shows 10 consecutive response–recovery cycles of COF_TB_ for 500 ppm C_2_H_6_O_2_, which demonstrates the high reproducibility of the COF_TB_-based sensor under 500 ppm C_2_H_6_O_2_ conditions. Figure 5d shows a line graph of the fluctuation in 10 stable response–recovery cycles, and it can be seen that the fluctuation in 10 response cycles is small, again emphasizing the good experimental reproducibility. These results further demonstrate the potential of COF_TB_ in C_2_H_6_O_2_ detection applications.

Ambient humidity is also an important factor affecting the performance of gas sensors. The response of COF_TB_ to different relative humidity (RH) was tested, as shown in Figure S4. With the increase in RH, the response of COF_TB_ to RH shows an increasing trend and reaches 1.73k% at 95% RH, which is about 12.6% of the response to 500 ppm C_2_H_6_O_2_. Meanwhile, according to the test method in Figure 5e, we also tested the response curve of the sensor under common humidity (33% RH and 65% RH), as in Figure 5f. The response value decreased by 8.5% at 65% RH compared to 33% RH, but it did not have much effect on the ability of COF_TB_ to detect C_2_H_6_O_2_ under common humidity.

Long-term stability is crucial for the lifetime of gas-sensitive materials, and the response curves of COF_TB_ to 500 ppm C_2_H_6_O_2_ at 0, 25, and 50 days are shown in Figure S5. Figure S5 shows that there is a decreasing trend in the response value of the samples to C_2_H_6_O_2_, which decreased by 7.4% after 25 days and 14.9% after 50 days, but this had little effect on the detection performance of the samples. Hydrolysis of the imine bonds in the samples over time may be the cause of this phenomenon, where the breakage of the imine bonds causes changes in the internal structure of the material, leading to the destruction of the original conductive structure [34] and, thereby, increasing resistance. Moreover, the hydrophilic groups in the materials make the COF materials hydrophilic, which may also accelerate the hydrolysis of imine bonds [35]. It may be possible to improve the long-term stability of COFs and their resistance to humidity by adding hydrophobic functional groups [36].

Table 1 summarizes the recent studies of various C_2_H_6_O_2_ sensors. By comparing the different metrics, it can be seen that COF_TB_ has obvious advantages in the detection of C_2_H_6_O_2_: COF_TB_ was synthesized under milder conditions, showed excellent selectivity and response to C_2_H_6_O_2_ at room temperature, and had a low detection limit, demonstrating a high sensitivity. These comprehensive indices prove the potential application value of COFs as gas-sensitive materials.

### 3.3. Analysis of Sensing Mechanism

As shown in Figure S6, the linear current–voltage (I–V) indicates ohmic contact between the COF_TB_ and the electrode [42,43]. The main factors affecting the sensitivity and selectivity of COF_TB_ materials with n-type semiconductor properties should be attributed to the large specific surface area, hydrogen bonding interactions and high defects [25,33,44,45].

Firstly, COF_TB_ has a specific surface area of 10.04 m^2^g^−1^ and a spherical rough surface, which provides a large number of active sites for adsorption. The average pore size of COF_TB_ is 19.8 nm, which provides a large number of channels for the diffusion and transport of gas molecules [46]. Numerous pores allow the COF_TB_ sample to better bind with gas molecules, which improves the sensing response.

**Figure 6:**
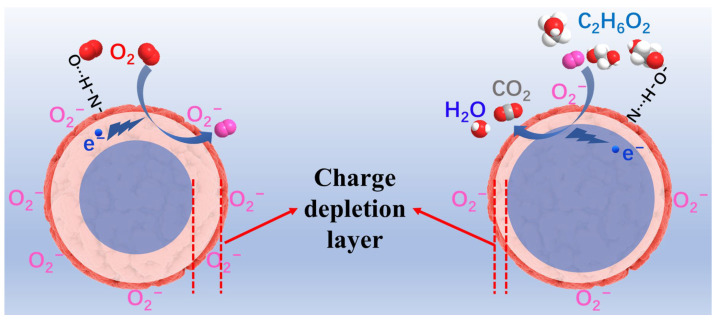
Sensing mechanism of sensor based on COF_TB_ to C_2_H_6_O_2_ vapor.

Secondly, the presence of a large number of imine bonds and amino functional groups in COF_TB_ may make it easier for different gas molecules to be adsorbed onto the surface of COF_TB_ nanospheres through hydrogen bonding (Figure 6). The electron depletion layer (*L*) is positively related to the oxygen ion concentration (*N_t_*) on the surface of sensing materials and inversely related to the charge carrier concentration (*N_d_*) of the sensing material, as shown in Equation (1). The change of *L* causes a change in the resistance of the sensing material, and the greater the change of *L*, the better the gas sensing performance of the sensing material.

(1) $$L\propto \sqrt{\frac{N_{t}^{2}}{N_{d}^{2}}}=\frac{N_{t}}{N_{d}}$$

When the material is exposed to ambient air, hydrogen bonds are formed between the large amount of N-H exposed on the surface of the COF_TB_ and the O_2_ in the air, thus adsorbing the O_2_ on the surface (Figure 6). Due to the strong electronegative nature of oxygen atoms, oxygen molecules capture electrons from the surface of the COF_TB_ material, as shown in Equation (2), which is converted into chemisorbed oxygen-negative ions at room temperature. Because the temperature is lower than 100 °C, oxygen molecules which capture an electron are then converted into O_2_^−^, as shown in Equation (3) [33].

O_2_ (gas) → O_2_ (ads) (2)

O_2_ (ads) + e^−^ → O_2_^−^ (ads) (<100 °C) (3)

Due to the adsorption of O_2_ molecules, the electrons of the COF_TB_ material were taken away, the carrier concentration decreased, and the *L* increased [34]. The increasing *L* hinders the electron transport in the material and causes the resistance of the material to rise [35]. When the COF_TB_ material is exposed to C_2_H_6_O_2_ vapor, the N atoms of the COF_TB_ material form hydrogen bonds with the -OH of C_2_H_6_O_2_, which makes it easier for C_2_H_6_O_2_ molecules to adsorb onto the surface of the COF_TB_ material. The C_2_H_6_O_2_ molecules and O_2_^−^ on the surface of the COF_TB_ material undergo the reaction shown in Equation (4). Electrons are released and return to the conduction band of COF_TB_ material; the *L* and the resistance of the material decrease [17]. At the same time, C_2_H_6_O_2_ has a stronger electron-donating ability than other gases [4], which also makes the COF_TB_ material have a higher response to C_2_H_6_O_2_.

2 C_2_H_6_O_2_ + 5 O_2_^−^ → 4 CO_2_ + 6 H_2_O + 5e^−^ (4)

Finally, the disordered structure formed during the synthesis of COF_TB_ improves the conductivity of the material. Crystalline COFs have poor gas-sensitive properties due to the presence of many crystal boundaries, which prevent the migration of carriers. Conversely, disordered COFs form a three-dimensional conductive network due to the lack of such boundaries, which facilitates the transfer of carriers through the material and contributes to the improvement in the gas-sensitive properties of the material [25]. The reason for the poor crystallinity of COF_TB_ is related to the amount of catalyst and the mechanism of synthesis of imine COFs. The synthesis of imine COFs is a dynamic and reversible process. Firstly, a large number of amorphous structures are formed rapidly by dehydration reactions between monomers, followed by a slow reorganization into crystalline structures by reversible reactions of imine bonds [34], in which appropriate amounts of catalyst and water are required. However, a large amount of catalyst was added at one time during our experiments, which led to the rapid formation of many disordered amorphous structures. The excess catalyst and less water used inhibited the reversible reaction of imine bonding [23], which made it difficult for the material to be transformed into a crystalline material, so that the COF_TB_ material with disordered structure and small specific surface area was obtained [47] as indicated by the results of the XRD, TEM and BET of the material. In conclusion, the synergies of large specific surface area, hydrogen bonding interactions and high defects determine the high selectivity and sensitivity of COF_TB_ towards C_2_H_6_O_2_.

## 4. Conclusions

In this work, COFs with highly defective amorphous structure were synthesized using catalysts at room temperature, and the COF_TB_ nanospheres with rough surfaces had high specific surface area. The rapid synthesis method using catalysts greatly increased defects, improved the electrical conductivity of COF materials and enhanced the sensitivity and selectivity of COF_TB_ to C_2_H_6_O_2_ at room temperature, with a theoretical LoD of 40 ppb. Moreover, COF_TB_ maintained good sensitivity to C_2_H_6_O_2_ vapor at room temperature under common humidity environments, showing high sensing stability. This study expands the application of COF materials and provides a C_2_H_6_O_2_ sensor that is functional at room temperature. However, the sensing performances of COF_TB_ material still need to be improved. For example, COF_TB_ is susceptible to high humidity, and it may be possible to improve the humidity resistance of the COF_TB_ material by adding hydrophobic functional groups to further expand the range of humidity at which the COF_TB_ material can be applied.

## Figures and Tables

**Table 1:** Various glycol sensors reported in recent literature.

Materials	Concentration (ppm)	Response	Preparation Method	LoD (ppb)	Temp (°C)
ErFeO_3_ [32]	100	15.8 ^b^	Electrostatic spinning	35	230
ZnO/ZnCo_2_O_4_ [13]	100	15.63 ^b^	Hydrothermal method	1590	160
ZnO/rGO [14]	100	277 ^b^	Hydrothermal method	1000	200
CuO/Co_3_O_4_ [4]	100	6.3 ^b^	Hydrothermal method	-	130
SmFeO_3_ [37]	100	18.19 ^b^	Electrostatic spinning and calcination	-	240
NTO [38]	100	160.72 ^b^	Chemical vapor deposition	472	125
G-NiO-ZnO [39]	100	142 ^b^	Hydrothermal method	-	140
La-doped ZnSnO_3_ [40]	100	1488.79 ^b^	Hydrothermal method	200	140
(SEMCs)/SnO_2_ [41]	100	132 ^b^	Carbonization and activation	4.8	160
This work	500	13,880 ^a^	Normal temperature catalyst synthesis	40	RT

^a^ (*R_gas_* − *R_air_*)/*R_gas_* × 100%; ^b^ *R_gas_*/*R_air_*.

## Data Availability

The data presented in this study are available on request from the corresponding author.

## Supplementary Materials

The following supporting information can be downloaded at: https://www.mdpi.com/article/10.3390/nano13243103/s1, Figure S1: Target gas preparation diagram; Figure S2: Response-recovery curves of COF_TB_ toward 20 different atmospheres (500 ppm); Figure S3: Histogram of averages and error bars based on three responses; Figure S4: (a–c) Sensing curves of COF_TB_ for different humidity levels; Figure S5: Long-term stability of COF_TB_ to 500 ppm C_2_H_6_O_2_; Figure S6: I–V curves of COF_TB_.
