# Supplementary material for: High-Performance Ethylene Glycol Sensor Based on Imine Covalent Organic Frameworks
Source: Nanomaterials (Basel). 2023 Dec 8;13(24):3103. doi: 10.3390/nano13243103 (PMC10745960; doi:10.3390/nano13243103)
Supplement: Supplementary file 1 [file nanomaterials-13-03103-s001.zip › nanomaterials-2741374-supplementary.pdf]

Supplementay Materials

Shiwei Liu [1], Guojie Zhang [1], Weiyu Zhang [1], Ning Tian [1,*], Qihua Sun [1] and Zhaofeng Wu [1,2,*]

[1] Xinjiang Key Laboratory of Solid-State Physics and Devices, Urumqi 830046, China; 107552103375@stu.xju.edu.cn (S.L.); 107552103366@stu.xju.edu.cn (G.Z.);
107556522113@stu.xju.edu.cn (W.Z.); sunqh@xju.edu.cn (Q.S.)
[2] School of Physics Science and Technology, Xinjiang University, Urumqi 830046, China
* Correspondence: tn@xju.edu.cn (N.T.); wuzf@xju.edu.cn (Z.W.)

Materials and Reagents:

1,3,5-tris(4-aminophenyl) benzene (TAPB (99.5%)) and 4,4'-Biphenyldicarbaldehyde (BPDA (99.5%)) were purchased from Shanghai Tensus Biotech Co., Ltd. (Shanghai, China). Benzaldehyde (C_7H_6O (99%)), DMSO (99%), NMP (99%), toluene (C_7H_8 (99.5%)) and acetonitrile (C_2H_3N (99%)) were purchased from Shanghai Boer Chemical Reagents Co., Ltd. (Shanghai, China). Ethyl acetate ($C_4H_8O_2$ (99.5%)) and methanol (CH_4O (99.5%)) were purchased from Tianjin Xinbote Chemical Co., Ltd. (Tientsin, China). 1,4-Dioxane ($C_4H_8O_2$ (99.5%)) was purchased from Tianjin Zhongyuan Chemical Reagent Co., Ltd. (Tientsin, China). 1,3,5-Trimethylbenzene (C_9H_{12} (97%)) was purchased from Shanghai Mclean Biochemical Technology Co., Ltd. (Shanghai, China). scandium(III) trifluoromethanesulfonate $Sc(OTf)_3$ (99%) was purchased from Beijing Biotech Pharmaceutical Co., Ltd. (Beijing, China). Formaldehyde (CH_2O (37%)), ethylene glycol (C_2H_6O (99.9%)), acetone (C_3H_6O (99.9%)), NH_3 (25%), ethylene glycol ($C_2H_6O_2$ (99%)) and aniline (C_6H_7N (99.9%)) were purchased from Sinopharm Chemical Reagent Co., Ltd. (Beijing, China). Hydrogen peroxide (H_2O_2 (30%)) HCl (37%)，Hexane (C_6H_{14} (99.5%)) and HNO_3 (65%) were purchased from Aladdin Reagent Co., Ltd. (Shanghai, China). Phenol (C_6H_6O (99%)) was purchased from Tianjin Damao Chemical Reagent Factory (Tientsin, China). Trimethylamine solution (C_3H_9N (33%)) was purchased from Tianjin Kwangfu Fine Chemical Industry Research Institute (Tientsin, China).

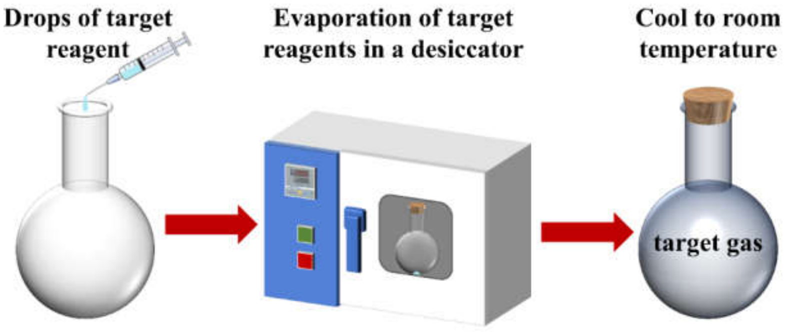

Figure S1. Target gas preparation diagram.

All target gases were prepared by thermal evaporation using the following equation:

$$Q = (V \times C \times M)/(22.4 \times D \times \rho) \times 10^{-9} \times (273 + T_R)/(273 + T_C)$$

where Q is the liquid volume of the target reagent (mL), V is the volume of the sealed experimental container (mL), C is the concentration of the prepared target atmosphere (ppm), M is the molecular weight of the target reagent, d is the purity of the target reagent (wt %), ρ is the density of the target reagent (g/cm^3), T_R is the ambient temperature, and T_C is the actual temperature inside the sealed experimental container. The target gas is prepared by pumping the target liquid into a sealed experimental vessel and heating it.

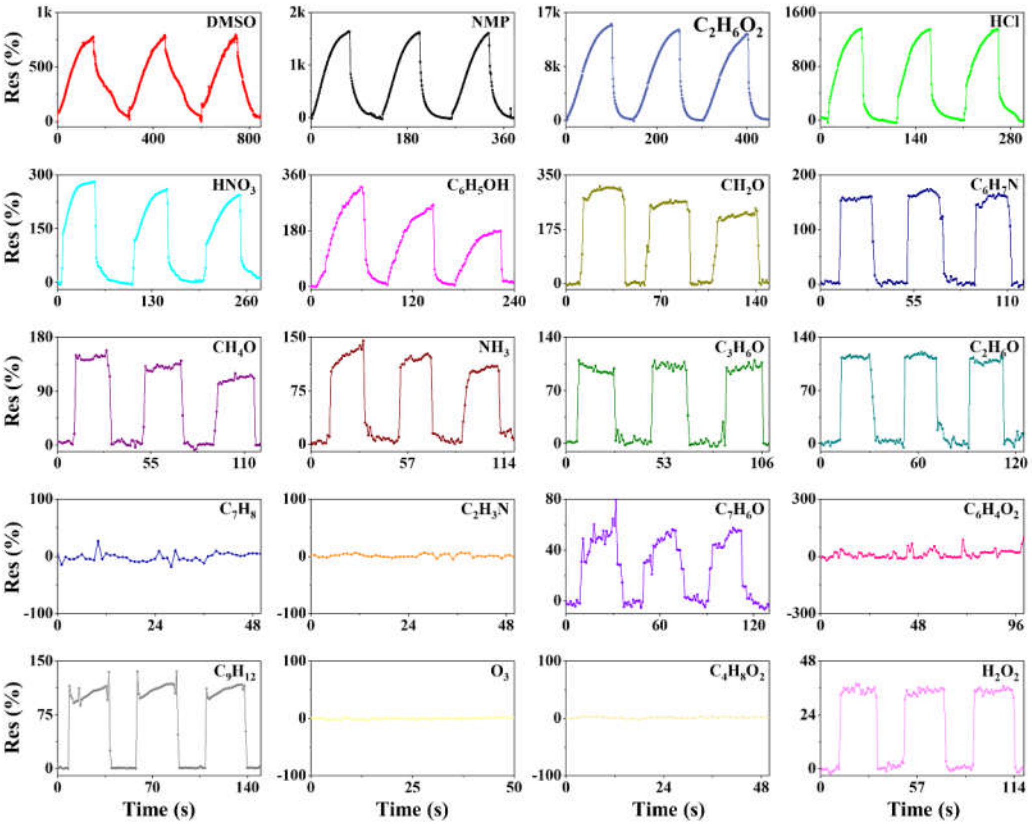

Figure S2. Response-recovery curves of COF_{TB} toward 20 different atmospheres (500 ppm).

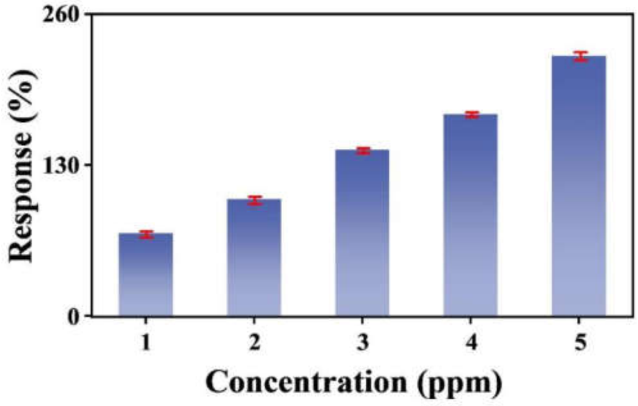

Figure S3. Histogram of averages and error bars based on three responses.

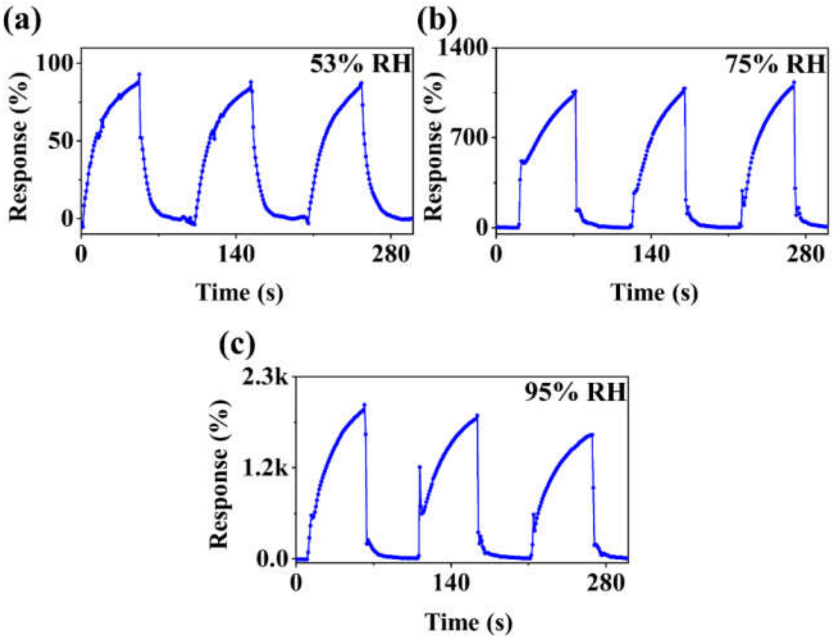

Figure S4. (**a–c**) Sensing curves of COF_{TB} for different humidity levels.

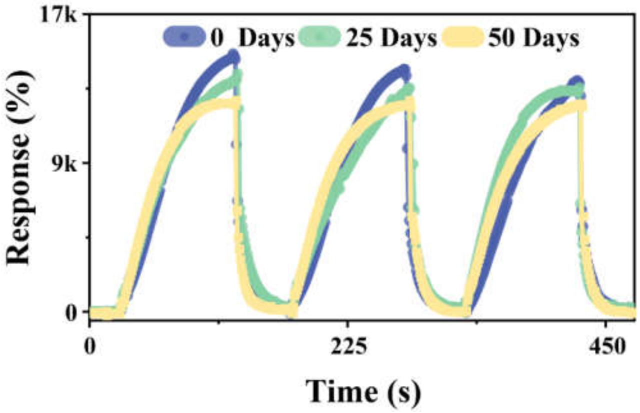

Figure S5. Long-term stability of COF_{TB} to 500 ppm $C_2H_6O_2$.

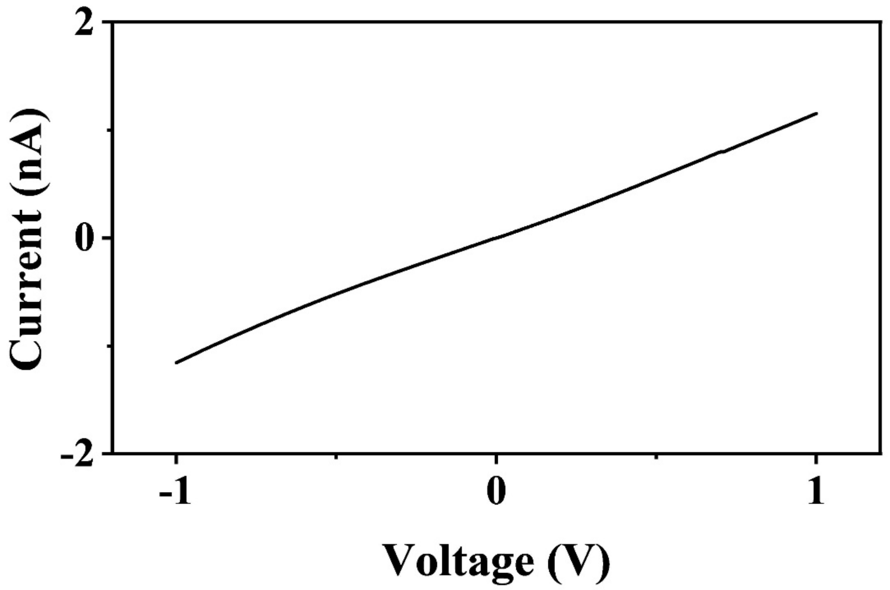

Figure S6. I-V curves of COF_{TB}.
